# First Experience in Korea of Stereotactic Partial Breast Irradiation for Low-Risk Early-Stage Breast Cancer

**DOI:** 10.3389/fonc.2020.00672

**Published:** 2020-04-29

**Authors:** Won Hee Lee, Jee Suk Chang, Min Jung Kim, Vivian Youngjean Park, Jung Hyun Yoon, Se Young Kim, Jee Ye Kim, Hyung Seok Park, Seung Il Kim, Young Up Cho, Byeong Woo Park, Yong Bae Kim

**Affiliations:** ^1^Department of Radiation Oncology, Breast Cancer Center, Yonsei Cancer Center, Yonsei University College of Medicine, Seoul, South Korea; ^2^Department of Radiology, Breast Cancer Center, Yonsei Cancer Center, Yonsei University College of Medicine, Seoul, South Korea; ^3^Department of Surgery, Breast Cancer Center, Yonsei Cancer Center, Yonsei University College of Medicine, Seoul, South Korea

**Keywords:** stereotactic partial breast irradiation, accelerated partial breast irradiation, breast cancer, Korean, feasibility studies, dosimetric outcomes, early toxicity

## Abstract

**Purpose:** Accelerated partial breast irradiation (A-PBI) in Korean women has been considered impracticable, owing to small breast volume and lack of high-precision radiotherapy experience. We present the first experience of stereotactic-PBI (S-PBI) with CyberKnife M6 to investigate feasibility of use and early toxicities in Korean women with early breast cancers.

**Materials and Methods:** A total of 104 breasts receiving S-PBI at our institution between September 2017 and October 2018 were reviewed. Patients were selected based on the American Society for Radiation Oncology (ASTRO), American Brachytherapy Society, American Society of Breast Surgeons, and Groupe Européen de Curiethérapie-European Society for Therapeutic Radiology and Oncology guidelines. A dose of 30 Gy in 5 fractions (NCT01162200) was used. Gold fiducials were routinely inserted near the tumor bed for tracking. Constraints regarding organs-at-risk followed the NSABP-B39/RTOG 0413 protocol.

**Results:** Median follow-up was for 13 months. Patients were categorized as “suitable” (71.2%) or “cautionary” (28.8%) according to 2017 the ASTRO guidelines. No tracking failure of inserted gold fiducials occurred. Median planning target volume (PTV) and PTV-to-whole breast volume ratio was 73.6 mL (interquartile range, 58.8–103.9 mL) and 17.0% (13.3–19.1%), respectively. Median PTV V_95%_, PTV D_max_, and ipsilateral breast V_50%_ were 97.8% (96.2–98.8%), 105.3% (104.2–106.4%), and 35.5% (28.3–39.8%), respectively. No immediate post-S-PBI toxicity ≥ grade 2 was reported, except grade 2 induration in three breasts. All patients remain disease-free to date.

**Conclusion:** The first use of S-PBI in Korean women was feasible and safe for selected early breast cancer. Based on these results, we have initiated a prospective study (NCT03568981) to test S-PBI in whole-breast irradiation for low-risk early breast cancer.

## Introduction

Accelerated partial breast irradiation (A-PBI) has emerged as an alternative to whole- breast irradiation (WBI). Previous studies in patients with low-risk early-stage breast cancer show that rates of local recurrence after A-PBI are extremely low, and most cases are limited to the vicinity of the original tumor bed (1, 2). Several prospective randomized trials demonstrated that A-PBI is associated with a non-inferior ipsilateral breast tumor recurrence (IBTR) rate, excellent cosmesis, and low treatment-related toxicity compared to WBI; however, there are some variabilities in outcomes owing to use of different radiation techniques and patient selection criteria (3–6). However, while A-PBI has been widely adopted worldwide for low-risk early breast cancer patients, A-PBI adoption remains limited in South Korea. The “Patterns of practice” study revealed that the use of A-PBI is far from widespread in South Korea (7).

With advancements in high-precision radiotherapy techniques, stereotactic body radiation therapy has become an emerging option for early breast cancer, in the form of stereotactic A-PBI (S-PBI). Several Western institutions have shown that S-PBI is a safe and feasible treatment in patients with early breast cancer who meet strict criteria (8–10).

Given this background, we have implemented A-PBI in Korean women, and report here our first experience in South Korea of using S-PBI for low-risk early breast cancer. Our aim was to investigate the feasibility and early treatment toxicity profile of S-PBI in Korean women.

## Materials and Methods

### Patient Selection

We reviewed patients treated with S-PBI using CyberKnife M6 (Accuray Incorporated, Sunnyvale, CA, USA) at our institution between September 2017 and October 2018. Patients referred for radiotherapy after breast-conserving surgery for breast cancer were screened by radiation oncologists for suitability for S-PBI, based on consensus guidelines of the American Society for Radiation Oncology (ASTRO), American Brachytherapy Society (ABS), American Society of Breast Surgeons (ASBS), and Groupe Européen de Curiethérapie-European Society for Therapeutic Radiology and Oncology (GEC-ESTRO) (11–14). Patients with invasive lobular carcinoma were eligible for S-PBI, as ASBS and ABS guidelines accept all invasive subtypes, whereas ASTRO and GEC-ESTRO guidelines accept invasive lobular carcinoma as “cautionary” and “intermediate risk,” respectively. In our institution, invasive lobular carcinoma with no multiplicity found in preoperative image and surgical pathology, and satisfying other criteria in the guidelines were eligible for S-PBI. Low risk breast cancer patients in this study were defined as patients satisfying the criteria of all of the above guidelines. These low risk patients were preferentially selected for S-PBI. Physicians explained expected benefits and risks of S-PBI in contrast to conventional WBI to these selected patients, and S-PBI was given to those only who agreed the treatment. Updates to guidelines during the course of the study were applied immediately (15, 16). Ultimately, patients categorized as “suitable” as well as “cautionary” according to ASTRO guidelines were included in the study.

Patients who experienced surgical complications, had positive resection margins, were younger than 45 years, or had multicentric tumors were ineligible for S-PBI. Only patients who had a follow-up period of longer than 6 months were included in this study. All patients diagnosed with breast cancer were evaluated preoperatively using breast magnetic resonance imaging (MRI), ultrasonography, and mammography.

### Fiducial Insertion and Simulation

S-PBI performed with CyberKnife M6 tracked gold fiducials inserted near the tumor bed as fiducial markers. At commencement of the study in September 2017, the gold fiducials were routinely inserted, with three gold fiducials inserted into patients' breasts at a 1 cm margin from the postoperative tumor cavity under ultrasonographic guidance. Upon insertion, the fiducials were placed in a non-coplanar position with respect to the radiographic orthogonal images of the CyberKnife M6, and the greatest possible extent of angular separation was aimed for. Mammography was performed immediately after insertion to confirm the presence of the gold fiducials, and simulation computed tomography (CT) was carried out at least 1 week later to minimize the effect of fiducial migration (17). Non-contrast 1 mm cut CT images were obtained, with the surgical scar marked by a radiopaque angiocatheter. Vac-Lok (CIVCO Radiotherapy, Coralville, IA, USA) devices were used to immobilize patients in the supine position with arms placed overhead (Figure S1).

### Treatment Planning

**Figure 1:**
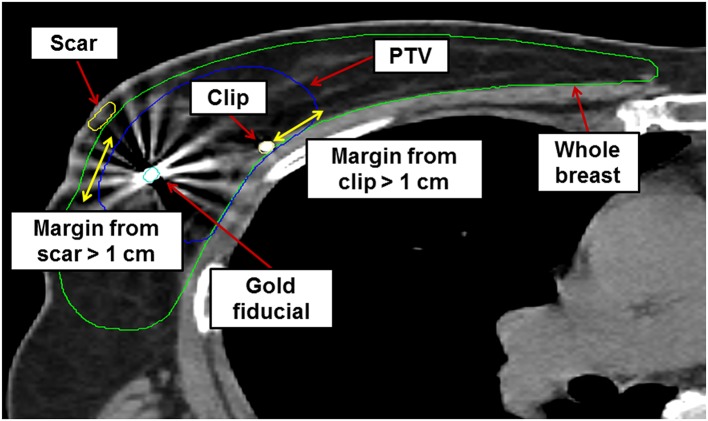
Example of axial cut image showing target delineation for stereotactic accelerated partial breast irradiation in a sample patient. PTV, planning target volume.

Ct images were imported into MIM software (MIM Software Inc., Cleveland, OH, USA) for target delineation. The surgical tumor cavity was identified based on pre- and postoperative images, surgical clips, and the incisional scar. The clinical target volume (CTV) was defined as a uniform 1 cm margin expansion from the tumor cavity, excluding the skin and chest wall. A margin of at least 5 mm from the breast skin surface was required. Chest wall structures, such as the pectoralis muscle or ribs, were excluded from the CTV. We defined the planning target volume (PTV) as equal to the CTV, using a robotic stereotactic tracking system capable of real-time respiratory tracking. The ipsilateral breast, contralateral breast, skin, chest wall, both lungs, heart, left anterior descending coronary artery, esophagus, thyroid, and spinal cord were delineated as organs-at-risk. The contoured PTV and ipsilateral whole-breast volume were measured using MIM software. The PTV-to-whole-breast ratio (PTV/WB) was calculated for each breast. An example of target delineation for S-PBI is shown in Figure 1.

The prescribed dose was 30 Gy in 5 fractions, identical to that used in work reported by the University of Texas Southwestern (UTSW), which proved safe and feasible in their phase I study (NCT01162200) (10). Following this regimen, radiotherapy was delivered every other day. The S-PBI was planned such that the PTV receiving 95% of the prescribed dose (V_95%_) would be over 95% of the total PTV, and the maximum point dose (D_max_) allowed for the PTV was <107%. Constraints to organs-at-risk mostly followed those of the NSABP B-39/RTOG 0413 protocol: ipsilateral breast V_50%_ <60%, contralateral breast D_max_ <1 Gy, ipsilateral lung V_30%_ <15%, heart (right-sided lesions) V_5%_ <5%, and heart (left-sided lesions) V_5%_ <40%. Doses to the contralateral lung, skin, chest wall, and thyroid were also considered according to the NSABP B-39/RTOG 0413 protocol.

### Treatment and Follow-Up

Robotic stereotactic radiotherapy using the CyberKnife M6 with fiducial tracking was used in all patients. Before every treatment, orthogonal X-ray images (from 45 and 135° angles with respect to the surface) were acquired after patient setup to visualize and align the fiducials with those in the original orthogonal X-ray images. If only two fiducials were detectable, treatment required authorization from a radiation oncologist.

Patients were interviewed and examined by the treating physician during the course of therapy, followed by routine visits every 6–12 months after S-PBI. Routine surveillance consisted of medical interviews, breast examinations, and mammography, in addition to optional breast ultrasonography and MRI. Toxicity assessment was performed using the Harvard scale, and mainly included breast skin change and induration assessments. In addition, skin thickness was measured by assessing ultrasound images obtained before surgery and 6–12 months after radiotherapy (if available). Both the skin above the tumor bed and the skin of the opposite quadrant of the ipsilateral breast (at least 5 cm away from the tumor bed) were measured at each time point.

For this study, we selected a cohort of 237 breasts that received WBI during the same period that the S-PBI was undertaken, for comparison of patient characteristics, toxicity, and skin thickness. All these breasts exhibited pathologically Tis or T1, node-negative breast cancers that received WBI of 40.05 Gy in 15 fractions, combined with a simultaneously integrated boost of 48 Gy in 15 fractions to the tumor bed by intensity-modulated radiation therapy (IMRT) after breast-conserving surgery.

### Ethical Statement

The hospital's institutional review board approved the retrospective review of S-PBI patients for this study (4-2019-0054). The necessity of written informed consent was waived due to the retrospective nature of the study, and the S-PBI patients in this study were not based on a protocol-based prospective study.

## Results

### Patient Characteristics

Between September 2017 and October 2018, 911 patients (922 breasts) were referred for radiotherapy after undergoing breast-conserving surgery. After screening, 103 patients (104 breasts; 11.3% of total referred breasts) received S-PBI. The median follow-up was 13 months (range, 6–21 months). The patient characteristics are summarized in Table 1. Among the total of 103 patients, the median age was 60 years (range, 46–85 years). Of the total of 104 breasts, 75 (72.1%) had invasive ductal carcinoma, with a median tumor size of 1.0 cm (range, 0.1–2.5 cm). Three patients had metastatic lymph nodes (1–2 sentinel lymph node metastases with no perinodal extension). The tumor grade was 1 or 2 in 97 breasts (93.3%). None of the tumors had lymphovascular invasion, and all had clear resection margins. All tumors except 1 were estrogen receptor-positive. The breasts were categorized as “suitable” (71.2%) or “cautionary” (28.8%) according to the updated 2017 ASTRO guidelines. The most common reason for classification as “cautionary” was extensive intraductal carcinoma of <3 cm (18 breasts). Compared to the pathologically Tis or T1, node negative WBI cohort, the S-PBI patients had younger age (*p* <0.01), lower tumor grade (*p* <0.01), less lymphovascular invasion (*p* <0.01), and more estrogen receptor positivity (*p* <0.01) (Table S1). The WBI cohort had trend toward more positive resection margin, larger tumor size, and more extensive intraductal component, although not statistically significant.

**Table 1:** Patient characteristics (per breast).

**Characteristic**	***N***	**%**
Age (years; median, range)	60 (46–85)	
Pathologic type
DCIS	15	14.4
IDC	75	72.1
Other	14	13.5
Tumor size (cm; median, range)	1.0 (0.1–2.5)	
N stage		
N0	101	97.1
N1	3	2.9
RM
Negative	104	100.0
Close or Positive	0	0.0
Grade
Grade 1	53	51.0
Grade 2	44	42.3
Grade 3	7	6.7
LVI
No	103	99.0
Yes	1	1.0
EIC
No	86	82.7
Yes	18	17.3
ER
No	1	1.0
Yes	103	99.0
ASTRO guideline category
Suitable	74	71.2
Cautionary	30	28.8
Unsuitable	0	0.0

*DCIS, ductal carcinoma in situ; IDC, invasive ductal carcinoma; N stage, nodal stage; RM, resection margin; LVI, lymphovascular invasion; EIC, extensive intraductal carcinoma; ER, estrogen receptor; ASTRO, American Society for Radiation Oncology*.

### Technical Feasibility of S-PBI

All 104 breasts had real-time tracking with inserted gold fiducials. All three inserted fiducials were trackable in 83 breasts (75.5%), and two of the three were trackable in 27 breasts (24.5%). There was no treatment interruption, reinsertion of fiducials, or re-simulation owing to tracking failure. The median treatment time was 33 min (range, 25–45 min) (Table 2).

**Table 2:** Treatment characteristics.

**Characteristic**	***N***	**%**
Number of tracked gold fiducials (among inserted)
3 fiducials	81	77.9
2 fiducials	23	22.1
Treatment time (min; median, range)	33 (25-45)	

### Dosimetric Outcomes

**Figure 2:**
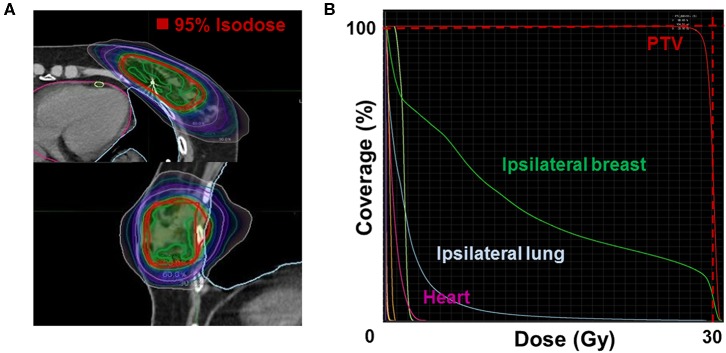
Example of **(A)** an isodose line (upper: axial; lower: sagittal) and **(B)** dose-volume histogram of a stereotactic accelerated partial breast irradiation plan that satisfies all dosimetric goals. PTV, planning target volume.

The median whole-breast volume was 481.1 mL [interquartile range (IQR), 375.1–646.4 mL], while the median PTV was 73.6 mL (IQR, 58.8–103.9 mL). The median PTV/WB was 17.0% (IQR, 13.3–19.1%). The dosimetric parameters for S-PBI in this study are shown in Table 3, while the PTV and PTV/WB in this study are compared to those found in other similar S-PBI studies in Table S2. The median PTV V_95%_ was 97.8% (IQR, 96.2–98.8%), and PTV D_max_ was 105.3% (IQR, 104.2–106.4%). The median ipsilateral breast V_50%_, ipsilateral lung V_10Gy_, and contralateral lung V_1.5Gy_ were 35.5% (IQR, 28.3–39.8%), 2.2% (IQR, 1.5–3.0%), and 0.0% (IQR, 0.0–0.0%), respectively. The median skin and chest wall D_max_ were 26.6 Gy (IQR, 25.5–28.0 Gy) and 29.8 Gy (IQR, 29.2–30.5 Gy), respectively. The mean dose for the heart was a median of 0.7 Gy (IQR, 0.5–1.2 Gy) and 0.4 Gy (IQR, 0.3–0.5 Gy), for left- and right-sided lesions, respectively. Figure 2 shows an example of an isodose line and dose-volume histogram of an S-PBI plan that successfully satisfied all dosimetric goals.

**Table 3:** Dosimetric outcomes of stereotactic partial breast irradiation.

**Dosimetric parameters**	**Median (interquartile range)**
PTV V_95%_	97.8% (96.2–98.8%)
PTV D_max_	105.3% (104.2–106.4%)
Ipsilateral breast V_50%_	35.5% (28.3–39.8%)
Contralateral breast D_max_	0.8 Gy (0.6–1.1 Gy)
Ipsilateral lung V_20Gy_	0.1% (0.0–0.3%)
Ipsilateral lung V_10Gy_	2.2% (1.5–3.0%)
Contralateral lung V_1.5Gy_	0.0% (0.0–0.0%)
Heart mean dose (left-sided lesions)	0.7 Gy (0.5–1.2 Gy)
Heart mean dose (right-sided lesions)	0.4 Gy (0.3–0.5 Gy)
Skin D_max_	26.6 Gy (25.5–28.0 Gy)
Chest wall D_max_	29.8 Gy (29.2–30.5 Gy)

*V_x%_, percentage of volume receiving X% of the prescribed dose; V_xGy_, percentage of volume receiving X Gy; D_max_, maximum point dose*.

### Physician-Rated Early Toxicity and Change in Breast Skin Thickness

**Figure 3:**
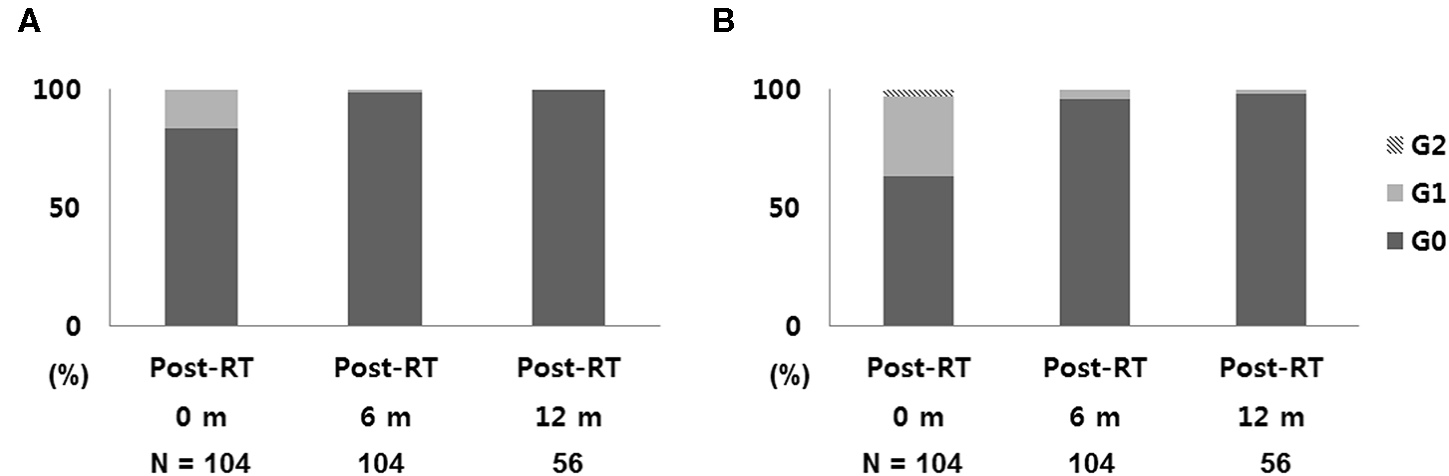
Early toxicity outcomes: **(A)** skin change and **(B)** breast induration after S-PBI. S-PBI, stereotactic partial breast irradiation; m, months.

After a median follow-up of 13 months, no IBTR, regional recurrence, or distant metastasis was detected in any of the patients. Figure 3 shows the toxicity data at the end of each follow-up period. Immediately after S-PBI, 87 breasts (83.7%) had no breast skin color change, and 66 (63.4%) had no palpable induration. No grade 2 or higher breast color change was reported, and grade 2 induration was observed in 3 breasts, which had persisted threesince immediately after the completion of surgery. After 6 months of follow-up, grade 1 color change and grade 1 palpable induration were noted in one and four breasts, respectively. Among the 56 breasts where follow-up of 1 year was reached, none showed color change and only one showed grade 1 induration. The WBI cohort showed similar results after 1 year of follow-up (135 breasts), as all except two breasts with grade 1 color change showed no color change, and two breasts had grade 1 induration. In terms of other treatment-related toxicities after S-PBI, one breast had grade 1 breast edema, and one breast had grade 2 breast cellulitis which was successfully managed with oral antibiotics. No rib fracture or radiation pneumonitis was noted after S-PBI.

**Figure 4:**
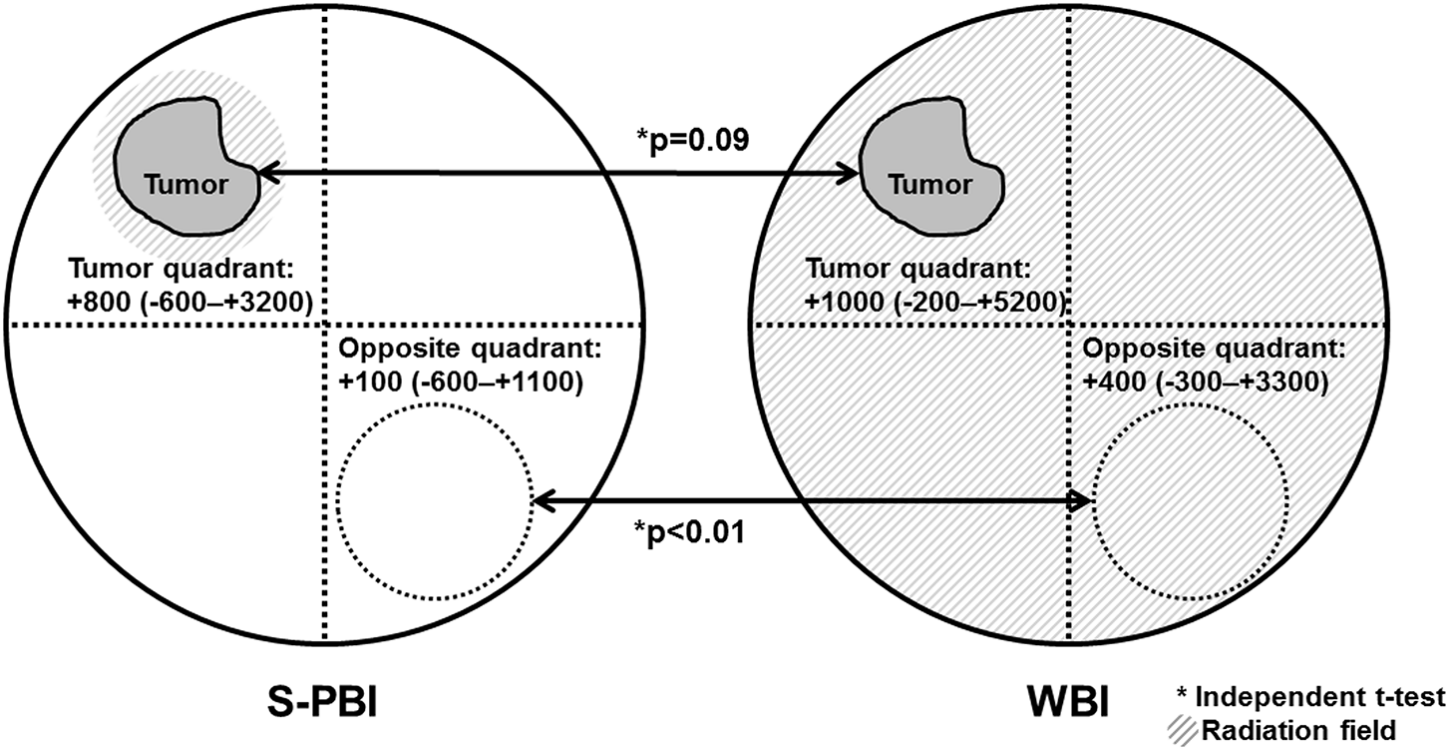
Changes in skin thickness after surgery followed by stereotactic partial breast irradiation (S-PBI) or whole-breast irradiation (WBI). Changes in skin thickness are defined as breast skin thickness before surgery, subtracted from breast skin thickness at 1 year after radiation. Values are presented in micrometers (range).

The change in skin thickness from before surgery to 6–12 months after radiotherapy was compared in the skin above the tumor bed and the skin of the opposite quadrant of the tumor bed (Figure 4). In S-PBI breasts, the median increase in skin thickness above the tumor bed was 800 μm (range, −600 to +3,200 μm), while skin of the opposite quadrant of the tumor bed in the ipsilateral breast increased by a median of 100 μm (range, −600 to +1,100 μm). In WBI breasts, the median increase in skin thickness above the tumor bed was 1,000 μm (range, −200 to +5,200 μm), while in the opposite quadrant of the tumor bed in the ipsilateral breast it increased by a median of 400 μm (range, −300 to +3,300 μm). Changes in skin thickness of the opposite quadrant were significantly smaller in the S-PBI group compared to the WBI group (*p* <0.01).

## Discussion

Our first experience of S-PBI revealed that it is a feasible and safe treatment in low-risk early breast cancer in Korean women. The high-precision radiotherapy technique showed excellent fiducial tracking abilities, with excellent dosimetric outcomes and minimal early toxicity, despite the relatively small breast volumes. To our knowledge, this is the first experience of S-PBI use in Korean women.

Over the last three decades, prospective trials using various techniques have demonstrated that A-PBI is non-inferior to WBI (3–6). However, only 4.7% of total radiation oncology facilities in South Korea use A-PBI (7). This could be due to several reasons. First, patient selection is limited, owing to the younger age distribution of breast cancer in South Korea compared to the Western hemisphere (18). In addition, even though many radiation oncologists have sufficient clinical experience in high-precision radiotherapy, they usually feel that it is unnecessary to apply such techniques because of the relatively small breast volumes and favorable clinical outcomes with conventional techniques. Lastly, but most practically, the Korean National Health Insurance (KNHI) program's reimbursement system, based on fraction number, has been a major obstacle to use of A-PBI.

Radical advances in IMRT and image guidance have provided a potential breakthrough for A-PBI, as shown in an Italian prospective trial (19). S-PBI, a further developed form of high-precision IMRT, has the potential to circumvent the limitations in Korean women. While A-PBI using conventional IMRT may carry risks owing to respiratory motion uncertainty, the novel high-precision technique of S-PBI addresses this with real-time motion tracking via fiducial markers, allowing minimal PTV margin expansion. We believe that S-PBI could provide a breakthrough for A-PBI in South Korea.

Our S-PBI was performed after careful patient selection. We considered all available A-PBI guidelines for selecting the patients. Only 6.2% of total breasts referred for radiotherapy were selected for S-PBI, and none were categorized as “unsuitable” according to the ASTRO guidelines. The results of strict patient selection are well described in Table S1, showing S-PBI patients bearing much more favorable clinicopathologic features. We were especially cautious when selecting patients aged 45–50 years, the gray zone among different guidelines (11–16). In this age group, only those without any relative contraindications were selected. As a result, despite the young age at which breast cancer frequently occurs in South Korea, as mentioned previously (18), we successfully managed to select an optimal group of Korean women for S-PBI.

We have also shown the technical feasibility of S-PBI in low-risk patients with early breast cancer. S-PBI was highly successful in terms of fiducial utilization, as no tracking failure occurred with routine gold fiducial insertion. All patients deemed eligible for A-PBI successfully underwent the procedure after fiducial insertion. The safety and efficacy of gold fiducial insertion for A-PBI has been well established by the UTSW, whose methods we followed (17). Moreover, the treatment time per fraction remained reasonable, compared to the UTSW S-PBI study (10). Each S-PBI treatment may be relatively longer than that for WBI, but the substantially shortened treatment total fraction ultimately saves both time and costs. Our first attempt at S-PBI in South Korea successfully proved that it is technically feasible in Korean women.

The dosimetric analyses in this study showed that S-PBI with minimal PTV expansion resulted in excellent dosimetric parameters in Korean women. During our initial S-PBI setup, we intended to set dose-volume constraints and define PTV based on the NSABP B-39/RTOG 0413 protocol, which establishes PTV as a uniform 1 cm expansion of CTV. However, we believed that modification of the definition of PTV was necessary, considering poor dosimetric outcomes in the ipsilateral breast in the Korean Radiation Therapy Oncology Group (KROG) 0804 study (20). Based on the high precision of S-PBI with successful fiducial tracking, and the preference of our surgeons for cavity shave margins over inked margins, we chose a much smaller PTV definition than that of the NSABP B-39/RTOG 0413 protocol.

As a result, not only were the ipsilateral breast dosimetric goals successfully satisfied in all breasts in our study, but the median ipsilateral breast V_50%_ in our study was 35.8%, much lower than that of the KROG study (20). Compared to the Western A-PBI reports (Table S2), the ipsilateral breast V_50%_ in our patients was as low as those in Western S-PBI studies (9, 21, 22), and dramatically lower than in A-PBI studies using three-dimensional conformal radiation therapy (3D-CRT), ranging from 42 to 49% (23–26). This could be explained by the substantial PTV margin expansion mandated by respiratory and setup uncertainties in 3D-CRT. Likewise, our delicate S-PBI planning achieved consistent dosimetric profiles compared to those observed in Western S-PBI studies in other organs-at-risk, without compromising PTV coverage or creating PTV hot spots (9, 21, 22). These results demonstrated that S-PBI could overcome the disadvantage of relatively small breast volumes in Korean women.

Early toxicities after S-PBI were minimal in our study. Although a few grade 1 or 2 palpable indurations due to surgery were observed, most patients did not experience any breast color change or palpable induration immediately after S-PBI. Any minimal color change or palpable induration had mostly recovered by the first follow-up visit after S-PBI, similar to the WBI group. Breast skin thickness is well known for its relationship with palpable induration, and radiotherapy is a well-known cause of thickening (27). In our study, the change in skin thickness after S-PBI appears to be limited to the tumor bed, in contrast to the diffuse skin thickening observed after WBI. These favorable toxicity profiles are comparable to those of the UTSW's identical dose cohort (10). They are also remarkably more favorable than those observed in prospective 3D-CRT A-PBI trials (6, 28, 29). In contrast to widespread concerns about hypofractionated radiotherapy in South Korea, S-PBI proved to be safe in terms of early toxicities in Korean women, despite small breast volumes.

Despite these promising findings, the KNHI reimbursement system still acts as a major barrier to S-PBI. The unreasonably low total income from S-PBI compared to WBI would ultimately prevent adoption of any form of A-PBI in Korean hospitals, even with sufficient proof of the technical feasibility and safety of S-PBI. Given the rapid developments in high-precision radiotherapy, the reimbursement system based on fraction size as a new parameter is a solution that should be actively considered (30). This could motivate hospitals to reduce loadings for patients, and ultimately provoke widespread use of A-PBI in Korean women.

Limitations of our study are its retrospective, single-institution nature, the limited number of patients, and the relatively short follow-up period. Longer follow-up may reveal whether these promising dosimetric outcomes and minimal early toxicity would translate into rare late toxicities and excellent cosmesis. However, we firmly believe that our first experience of S-PBI in Korean women will act as a cornerstone for widespread use of A-PBI in this population.

In conclusion, the first experience of S-PBI in Korean women demonstrated that it is a feasible and safe treatment for low-risk early breast cancer patients. Despite smaller breast volumes, outstanding dosimetric outcomes and successful fiducial tracking were achieved, with rare early toxicities. Based on this first experience in South Korea, we have initiated a prospective study (NCT03568981) to test S-PBI in terms of cosmesis and quality of life compared to WBI in early breast cancer.

## Data Availability Statement

The datasets generated for this study are available on request to the corresponding author.

## Ethics Statement

The studies involving human participants were reviewed and approved by Institutional Review Board of Human Reasearch Protection Center, Severance Hospital, Yonsei University Health System. Written informed consent for participation was not required for this study in accordance with the national legislation and the institutional requirements.

## Author Contributions

WL and JC contributed to data collection. WL wrote the initial manuscript with YK. YK had a huge role in setting up S-PBI in our department, with help from breast surgeons, who are JK, HP, SIK, YC, and BP. The breast surgeons, JK, HP, SIK, YC, and BP contributed to the discussion part where surgical margin and acute toxicity is described. VP, JY, and MK contributed in fiducial insertion during S-PBI in our institution and actively participated in the manuscript by giving ideas to measure skin thickness as an outcome for this study. SYK was the main dosimetrist of our S-PBI. Not only she created plans that successfully satisfied dosimetric goals, but also participated in setting up the CT-simulation and treatment process.

## Conflict of Interest

YK has received research funding from Accuray Inc. Sunnyvale, CA, USA. The remaining authors declare that the research was conducted in the absence of any commercial or financial relationships that could be construed as a potential conflict of interest.

## Supplementary Material

Figure S1 Example of simulation of stereotactic partial breast irradiation using Vac-Lok (CIVCO Radiotherapy, Coralville, IA, USA) for immobilization in supine position with arms placed overhead.

Table S1 Patient characteristics compared between stereotactic partial breast irradiation (S-PBI) and the whole breast irradiation (WBI) cohort^a^ (per breast).

Table S2 Comparison of PTV, PTV-to-whole-breast ratio, and irradiated ipsilateral breast volume among published studies of external beam accelerated partial breast irradiation.
