# Supplementary figures and images for: First Experience in Korea of Stereotactic Partial Breast Irradiation for Low-Risk Early-Stage Breast Cancer
Source: Front Oncol. 2020 Apr 29;10:672. doi: 10.3389/fonc.2020.00672 (PMC7201053; doi:10.3389/fonc.2020.00672)

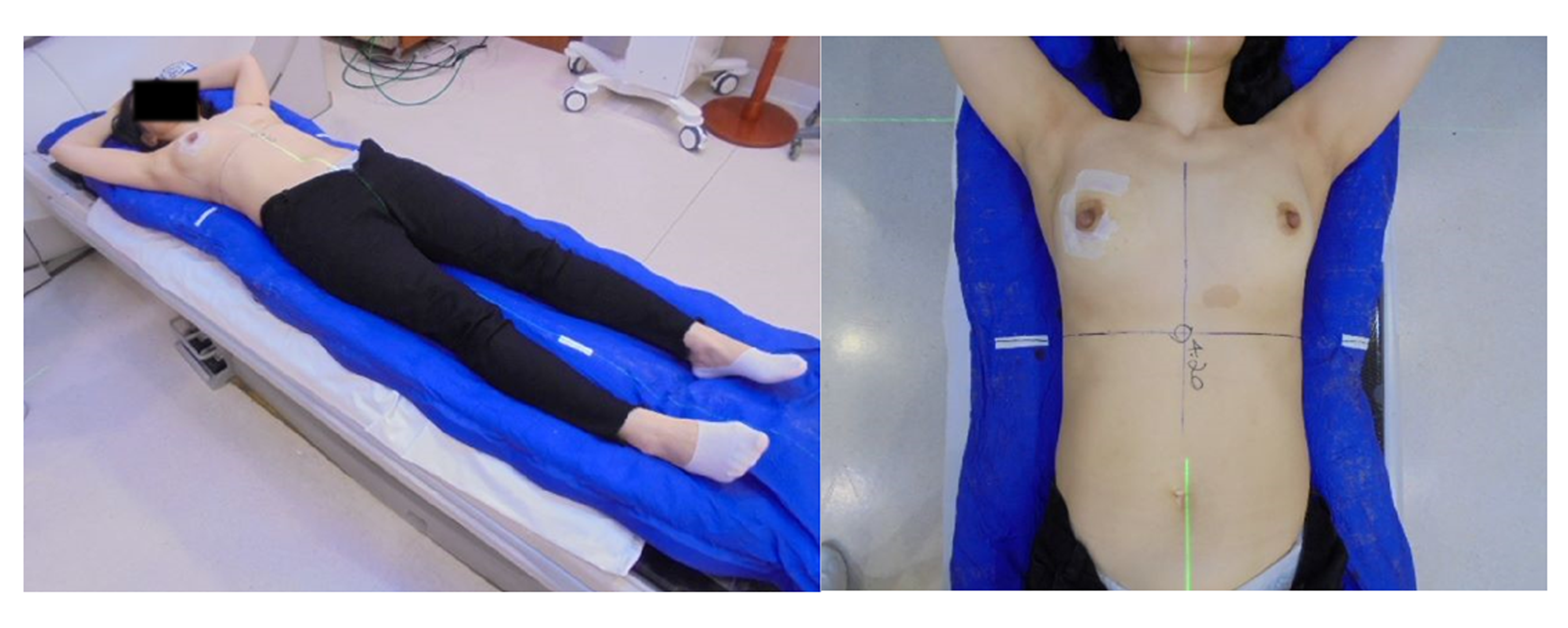

Supplement: Figure S1 — Example of simulation of stereotactic partial breast irradiation using Vac-Lok (CIVCO Radiotherapy, Coralville, IA, USA) for immobilization in supine position with arms placed overhead. [file Image_1.TIF]
