# Supplementary material for: First Experience in Korea of Stereotactic Partial Breast Irradiation for Low-Risk Early-Stage Breast Cancer
Source: Front Oncol. 2020 Apr 29;10:672. doi: 10.3389/fonc.2020.00672 (PMC7201053; doi:10.3389/fonc.2020.00672)
Supplement: Table S1 — Patient characteristics compared between stereotactic partial breast irradiation (S-PBI) and the whole breast irradiation (WBI) cohorta (per breast). [file Table_1.DOC]

**Table S1.** Patient characteristics compared between stereotactic partial breast irradiation (S-PBI) and the whole breast irradiation (WBI) cohort ^a)^ (per breast)

|  | WBI (n=237) | | S-PBI (n=104) | |  |
| --- | --- | --- | --- | --- | --- |
| Characteristic | N | % | N | % | p-value |
| Age (years; median, range) | 50 (21-79) | | 60 (46–85) | | <0.01 |
| Pathologic type |  |  |  |  | 0.13 |
| DCIS | 46 | 19.4 | 15 | 14.4 |  |
| IDC | 150 | 63.3 | 75 | 72.1 |  |
| Other | 41 | 17.3 | 14 | 13.5 |  |
| Tumor size ^b)^ (cm; median, range) | 1.2 (0.1-3.7) | | 1.0 (0.1–2.5) | | 0.37 |
| RM |  |  |  |  | 0.18 |
| Negative | 233 | 98.3 | 104 | 100.0 |  |
| Close or Positive | 4 | 1.7 | 0 | 0.0 |  |
| Grade |  |  |  |  | <0.01 |
| Grade 1 | 63 | 26.2 | 53 | 51.0 |  |
| Grade 2 | 108 | 45.6 | 44 | 42.3 |  |
| Grade 3 | 66 | 27.8 | 7 | 6.7 |  |
| LVI |  |  |  |  | <0.01 |
| No | 226 | 95.4 | 103 | 99.0 |  |
| Yes | 11 | 4.6 | 1 | 1.0 |  |
| EIC |  |  |  |  | 0.17 |
| No | 180 | 75.9 | 86 | 82.7 |  |
| Yes | 57 | 24.1 | 18 | 17.3 |  |
| ER |  |  |  |  | <0.01 |
| No | 62 | 26.2 | 1 | 1.0 |  |
| Yes | 175 | 73.8 | 103 | 99.0 |  |

DCIS, ductal carcinoma in situ; IDC, invasive ductal carcinoma; RM, resection margin; LVI, lymphovascular invasion; EIC, extensive intraductal carcinoma; ER, estrogen receptor. ^a)^The WBI patients selected for comparison all had pathologically Tis or T1 and node-negative breast cancer, treated by intensity-modulated radiation therapy with 40.05 Gy in 15 fractions with simultaneously integrated boost of 48 Gy in 15 fractions to the tumor bed during the same period. Tumor size represents size of invasive component in invasive histology, while it represents the total size of in situ component in ductal carcinoma in situ.

**Table S2.** Comparison of PTV, PTV-to-whole-breast ratio, and irradiated ipsilateral breast volume among published studies of external beam accelerated partial breast irradiation

|  | PTV volume (mL) | PTV-to-whole-breast ratio (%) | Ipsilateral breast V_50%_ (%) |
| --- | --- | --- | --- |
| Korean studies |  | | |
| Present study | 73.6 (21.4–211.2) | 17.0 (5.5–26.0) | 35.5 (12.3–52.6) |
| KROG 0804 (20) | 108.9 | 14.8 | - |
|  |  |  |  |
| Western studies |  |  |  |
| Winthrop/Swedish (22)^b)^ | 114^a)^ (39–241) | - | 29/26 (16–39) |
| Georgetown (9)^b)^ | 70^a)^ (35–142) | - | 31 (8–58) |
| UTSW (10)^b)^ | 87.4 (36.1–268.9) | 9.4 | - |
| Milan (19)^b)^ | 88.1 (32.3–238.8) | - | 28.7 ^a)^ (20.0–32.6) |
| University of Michigan (24)^c)^ | 185.8^a)^ (59.8–382.0) | - | 47.9 (22.7–79.1) |
| William Beaumont (25)^d)^ | 268.1^a)^ (61.8–623.0) | 17^a)^ | 49 (39–61) |
| Tufts (23)^d)^ | 296^a)^ (67–950) | 18 / 24^a) e)^ | 42 |
| NYU (26)^d)^ | 228^a)^ (57–1118) | 22^a)^ | 47 (23–75) |

PTV, planning target volume; V_x%_, percentage of volume receiving X% of the prescribed dose; KROG, Korean Radiation Oncology Group; UTSW, University of Texas Southwestern; NYU, New York University; ^a)^shown in mean value; ^b)^stereotactic partial breast irradiation; ^c)^intensity-modulated radiation therapy; ^d)^3-dimensional conformal radiotherapy; ^e)^excellent, good / fair, poor cosmesis, respectively. Values represented in median (range).
